# Particle Swarm Optimization and Multiple Stacked Generalizations to Detect Nitrogen and Organic-Matter in Organic-Fertilizer Using Vis-NIR

**DOI:** 10.3390/s21144882

**Published:** 2021-07-17

**Authors:** Mahamed Lamine Guindo, Muhammad Hilal Kabir, Rongqin Chen, Fei Liu

**Affiliations:** 1 College of Biosystems Engineering and Food Science, Zhejiang University, 866 Yuhangtang Road, Hangzhou 310058, China; guindo@zju.edu.cn (M.L.G.); 11913074@zju.edu.cn (M.H.K.); chenrq@zju.edu.cn (R.C.); 2 Department of Agricultural and Bioresource Engineering, Abubakar Tafawa Balewa University, Bauchi 0248, Nigeria; 3 Key Laboratory of Spectroscopy Sensing, Ministry of Agriculture and Rural Affairs, Hangzhou 310058, China

**Keywords:** PSO, multiple-stacked generalizations, Vis-NIR, nitrogen, organic-matter, organic fertilizer

## Abstract

Organic fertilizer is a key component of agricultural sustainability and significantly contributes to the improvement of soil fertility. The values of nutrients such as organic matter and nitrogen in organic fertilizers positively affect plant growth and cause environmental problems when used in large amounts. Hence the importance of implementing fast detection of nitrogen (N) and organic matter (OM). This paper examines the feasibility of a framework that combined a particle swarm optimization (PSO) and two multiple stacked generalizations to determine the amount of nitrogen and organic matter in organic-fertilizer using visible near-infrared spectroscopy (Vis-NIR). The first multiple stacked generalizations for classification coupled with PSO (FSGC-PSO) were for feature selection purposes, while the second stacked generalizations for regression (SSGR) improved the detection of nitrogen and organic matter. The computation of root means square error (RMSE) and the coefficient of determination for calibration and prediction set (R^2^) was used to gauge the different models. The obtained FSGC-PSO subset combined with SSGR achieved significantly better prediction results than conventional methods such as Ridge, support vector machine (SVM), and partial least square (PLS) for both nitrogen (R^2^p = 0.9989, root mean square error of prediction (RMSEP) = 0.031 and limit of detection (LOD) = 2.97) and organic matter (R^2^p = 0.9972, RMSEP = 0.051 and LOD = 2.97). Therefore, our settled approach can be implemented as a promising way to monitor and evaluate the amount of N and OM in organic fertilizer.

## 1. Introduction

Organic fertilizers (biofertilizers) are biodegradable and environmentally friendly, which makes better nutrient sources. The amount of nitrogen (N) and organic matter (OM) in biofertilizers may directly affect the physical and chemical properties of soil and play a positive role in crop development [1]. However, many industrial or even homemade biofertilizers fail to meet nutritional requirements, posing environmental risks. A high level of nitrogen (N) can evaporate into the atmosphere and result in serious environmental issues such as ammonia (NH_3_) and ozone (O_3_), limiting our ability to breathe, limiting visibility, and affecting plant growth. At the same time, excessive use of organic matter (OM) can result in the release of chemicals that delay plant growth and may promote the development of some unwanted plants. Therefore, rapid, cost-effective, and reliable determination is needed to improve local agricultural production, sustainability, and environmental protection.

Visible near-infrared spectroscopy (Vis-NIR) is an alternative tool for determining N and OM in biofertilizers as it offers more and better advantages than standard chemistry methods. It is a non-invasive procedure with high penetration of radiation beams, suitable for inline use, and requires no (minimum) sample preparation. It has been widely used for food-quality assessment and in various fields, such as agriculture [2], medicine [3], and environmental uses [4]. The Vis-NIR technique is built on the connection between electromagnetic radiation and the material within the 350–2500 nm wavelength range.

In the visible part of the electromagnetic spectrum (400–700 nm), molecular electron transitions prevail, while in the near-infrared field (700–2500 nm), overtone and molecular vibration variations of the mid-infrared spectrum dominate. Different properties may be correlated with the absorbed radiation, providing qualitative and quantitative information [5]. The visible range resulting absorption spectrum is mainly related to OM or oxides [6] and iron in minerals. Usually, the obtained spectra have extensive absorption features and overlapping bands, making them challenging to interpret. Therefore, it is crucial to execute a feature selection process to automatically pick the significant feature subset and apply a robust chemometrics technique to predict the different properties [7].

Feature selection is the process of minimizing the number of features in a dataset and making the model as compact as possible. It could be divided into three methods: wrapper, embedded, and filter method [8,9]. Wrapper and embedded methods are more efficient than the filter method but are computationally more costly. However, it is challenging for all three strategies to find an optimal feature subset using the objective function. Various search meta-heuristics were proposed to resolve this problem, e.g., genetic algorithms (GA) [10,11], particle swarm (PSO) [12], and ant colony optimization (ACO) [13]. These techniques have been proven to solve challenging computational problems.

PSO has been used with many machine-learning techniques for feature selection [14,15]. Still, there is no study in combining multiple-stacked generalization and PSO for feature selection to the best of our knowledge. Furthermore, our approach to combining Vis-NIR data, PSO, and two multiple-stacked generalizations to determine N and OM can be considered a novel strategy.

Recently, there have been a number of low-quality biofertilizers on the market that exceed the nutrient value requirements. This issue could jeopardize the long-term viability of agriculture and the environment.

The main goal of this research was to prove that Vis-NIR can evaluate N and O.M. in biofertilizers. The specific objectives of this research were to: (1) propose a novel way for best subset selection using an ensemble method (first multiple stacked generalizations for classification, FSGC) and PSO; (2) improve the detection of N and OM through the construction of a second ensemble method for regression (SSGR), and (3) evaluate the performance with conventional techniques.

## 2. Materials and Methods

### 2.1. Samples

Nine varieties of commercial organic fertilizers from various companies in China were used to conduct this experiment. The different varieties of fertilizers were made of earthworm manure (variety 1), fungi (variety 2 and 5), fermented sheep manure (variety 3), a mix between sheep fat, chicken manure, grass charcoal, and bean cake (variety 4), black chicken fat (variety 6), biochar (variety 7), fulvic potassium acid and tobacco powder (variety 8) and rice straw for variety 9.

Each variety was composed of 30 samples, which led to a total of 270 samples. Sample preparation was not conducted to keep the experiment original and practicable. The true concentration of organic matter (OM) and nitrogen (N) was measured by inductively coupled plasma optical emission spectrometry (ICP-OES). Samples were first weighed into TFM vessels, and 5 mL of nitric acid (HNO_3_) and 1 mL of hydrogen peroxide (H_2_O_2_) were added. Second, microwave digestion (MARS 6, CEM, Mathews, VA, USA) was used to digest the solution. After digestion, the solutions were mounted in a volumetric flask and diluted with deionized distilled water (H_2_O). Finally, the concentration of N and OM was calculated by ICP-OES (Optima 8000, PerkinElmer, Waltham, MA, USA). The statistics of O.M. and N concentrations in samples were listed in Table 1.

### 2.2. Visible Near-Infrared Spectroscopy (Vis-NIR) Measurements and Preprocessing

The Vis-NIR spectra were measured in ambient light using an ASD (Analytical Spectral Devices) FieldSpec3 spectrometer over the reflective domain of 350–2500 nm. The radiation was estimated at 1.4 nm interludes for the 350–1000 nm spectral area and m intervals for the 1000–2500 nm wavelength range by a spectrometer. Finally, the reflectance output to users was resampled with ViewSpecPro (Version 6.20 Malvern Panalytical Ltd., Malvern, UK).

Biofertilizer samples were placed in a Petri dish and then smoothed with a ruler to homogenize the surface [16]. Furthermore, A 50 W halogen lamp was hung directly above each experimental Petri dish. The spectrometer was then calibrated with a Spectralon (Malvern Panalytical Ltd., Malvern, UK) white plate once every 15 measurements. Each soil sample was repeatedly measured three times. Finally, 810 spectra were obtained and preprocessed with the Savitzky–Golay smoothing filter introduced in 1964 [17]. Apart from this smoothing method, any other one has been conducted.

### 2.3. Construction of the Multiple Stacked Generalizations

Stacked generalization is a method of combining numerous models in order to perform a classification task [18,19]. In this work, several stacked layers have been generated by assigning the final estimator to the stacked generalization, and various models such as SVM, L.R., KNN, R.F., MLP, NB, and Ridge were used. The best parameter settings of each model were obtained after performing parameter tuning optimization using a grid search cross-validation five folds. After comparing the accuracy and the running time for all the classifiers, the best four models were chosen to form the FSGC model.

- Support vector machine (SVM): this selects important instances to create a separating surface between data instances [20]. After performing a grid search, the obtained optimized learning parameters for SVM were C = 1, gamma = 0.01, kernel = rbf.
- Logistic regression (L.R.): this is used to define variables and illustrate a relationship between one dependent variable and one or more independent variables [21]. After examining a wide collection of options using a grid search, the best parameter settings were C = 100, penalty = l2, solver = newton-cg.
- K-nearest neighbor (KNN): this model is among the simplest of all ML methods, and it is used to define and for prediction. The best hyperparameters for this model were metric = Euclidean, n neighbors = 1, weights = uniform [22].
- Random forest (RF): this approach is better than a single decision tree since it eliminates over-fitting by averaging the answer. The optimized learning parameters of SVM were max_depth = 80, max_features = 2, min_samples_leaf = 3, min_samples_split = 10, n_estimators = 200 [23].
- Multilayer perceptron (MLP): this is made of three layers of nodes that use a nonlinear activation function [24]. The best parameters for this model after running 5 folds were activation = tanh, alpha = 0.0001, hidden_layer_sizes = 103,010, learning_rate = constant and solver = adam.
- Gaussian naïve Bayes (N.B.): this algorithm focuses on the Bayes theorem, and it is used for classification, but it has a high versatility when the dimensions of the inputs are large. Complex classification issues can also be tackled using the Naive Bayes Classifier [25].
- Ridge: this is a straightforward linear regression that applies a slight degree of bias to obtain a significant decrease in volatility. Using grid search cross-validation, it has been found that the best bias value is 0.1.

### 2.4. Feature Selection

When using wrapper methods for feature selection, the significant problems are selecting the model and selecting a proper set of parameters for the used model. These problems can be surpassed by choosing an adequate model and optimizing the parameters. Thus, this section presents a new wrapper method by combining multiple-stacked generalizations and a PSO algorithm. The proposed method was called PSO-FSGC and can be divided into the following steps:

- Step 1: Create the multiple-stacked generalization classifier.

The FSGC presented in Section 2.4 was used. Each model in FSGC was optimized by performing grid search hyperparameter tuning.

- Step 2: Evaluate each particle in the swarm.

In this step, the stacked generalization created above was generated to find the feature subset corresponding to the particle position.

- Step 3: Verification of the best values of swarm and particle.

The following two equations achieve the verification:

(1) $$x_{i}^{pb}\leftarrow x_{i\;}\;if\;f\left(x_{i\;}\right)\;>f\left(x_{i}^{pb}\right)$$

(2) $$x^{sb}\leftarrow x_{i\;}\;if\;f\left(x_{i\;}\right)\;>f\left(x^{sb}\right)$$

With $x^{pb}$ correspond to the position of the particle that had the best fitness f and $x^{sb}$ is the best swarm.

- Step 4: Update velocities of the movement in the search space.

The update must take into consideration the performance of the own particle and the swarm. The velocity is updated as follows:

(3) $$v_{ij}\leftarrow wv_{ij}+c_{1}q\left(\frac{x_{ij}^{p_{b}}-x_{ij}}{\Delta t}\right)+\;c_{2}r\left(\frac{x_{j}^{s_{b}}-x_{ij}}{\Delta t}\right)$$

The term w is the inertia weight, and it controls the influence of the previous velocity in the new velocity. Δt is the time step of each iteration. $c_{1}$, $c_{2}$ are called cognitive and social components. The first tests the degree of self-confidence of a particle, while the second depends on the capacity of the swarm to identify better candidate solutions. q and r are uniform random numbers ∈ [0, 1].

- Step 5: Update the position of the particle by using the logistic function of the velocity.

Thus, the particle position is calculated for each variable by:

$S\left(v_{ij}\right)$ is the sigmoid function defined as:

(4) $$x_{ij}\leftarrow \left\{\begin{array}{c} 0,\;\;\;\;\;\;if\;r>S\left(v_{ij}\right)\\ 1,\;\;\;\;\;\;\;\;\;\;\;otherwise \end{array}\right.i=1,\ldots ,N,\;j=1,\ldots ,N$$

(5) Svij =11+evij

- Step 6: Continue the iterative process.

Return to Step 2 if convergence or iteration limit is not achieved. There is a possibility that premature convergence arises if the velocity reaches a high value (5) and (6). In that case, we apply the reset swarm that could allow each particle to adjusts its position according to two values: its own best solution $x^{pb}$ and the swarm best solution $x^{sb}$ [26,27].

### 2.5. Model Evaluation and Software

The performances were estimated according to their predictions. Thus, the computation of root means square error of calibration set (RMSEC), root means square error of prediction set (RMSEP) and limit of detection (LOD) helped gauge the performance. Furthermore, to measure other variables’ detectability, the coefficient of determination for calibration (R^2^c) and prediction (R^2^p) were computed. Generally, a good model should have high values of R^2^. The Savitzky–Golay smoothing was done with viewSpec software, the computation of the different algorithms was made using python with sci-kit-learn and PySwarms library, and Origin 2021 served to design the graph.

## 3. Results

### 3.1. Spectra Analysis and Visualization

**Figure 1:**
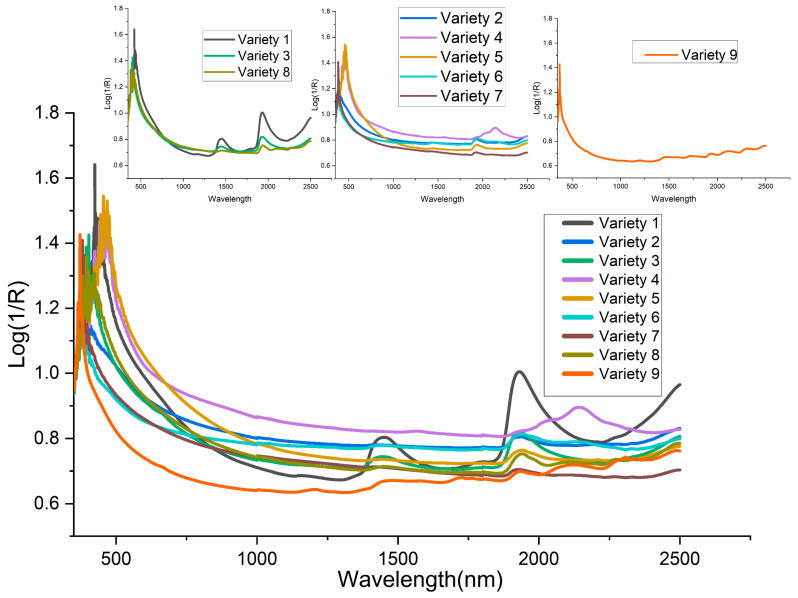
Average spectra of the nine varieties of organic fertilizer.

**Figure 2:**
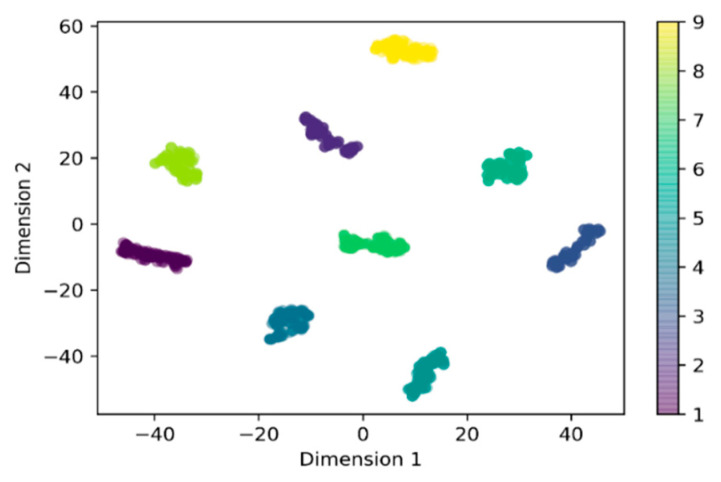
Visualization of the nine varieties in two dimensions using t-distributed stochastic neighbor embedding (t-SNE).

The average spectra of the nine varieties of biofertilizer samples were presented in Figure 1. Almost all the varieties have a similar profile curve. The loading density and particle size were attributed to the broad disparity of baseline shifts in the spectra. It can be observed that all the spectra show absorption at the range 1887−2200 nm. For variety 1, 3, 8, 9, peaks were observed at 1388–1549 nm, while three others peaks (844, 1733, and 2310 nm) were only observed for variety 9. T-distributed stochastic neighbor embedding (t-SNE) was used to map all the features into two-dimensional space to visualize the nine varieties of samples. The Vis-NIR datasets structure was preserved during the transformation, and it was seen that nine distinct clusters were formed (Figure 2). The visualization of t-SNE further proved the probability of classifying different biofertilizers using the Vis-NIR technique.

### 3.2. Models for the Stacked Generalization

**Figure 3:**
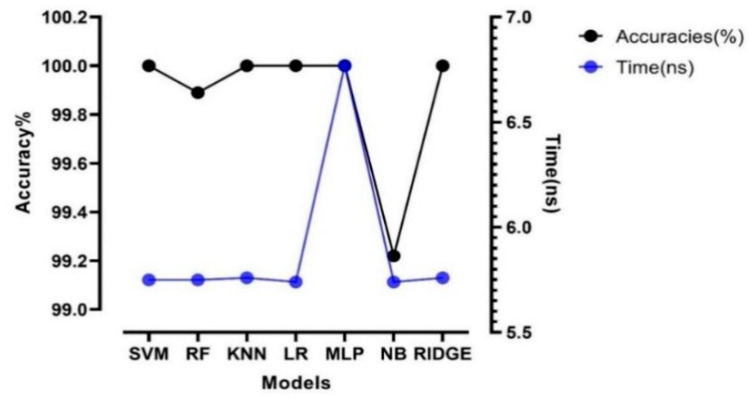
Accuracy and computing time of different algorithms.

The choice of the models and their best parameters are essential when creating a stacked generalization. Several models such as SVM, R.F., NB, KNN, L.R., MLP, and Ridge were computed with grid search hyperparameter tuning. The sample was split using cross-validation five folds. The obtained results showed that SVM, KNN, L.R., MLP, and Ridge outperformed with an accuracy of 100% (Figure 3). However, the running time comparison illustrated that MLP uses more time to be computed with 6.77 ns ± 0.00577 ns per loop, contrary to other models. (Figure 3). The comparison of both accuracy and time showed that SVM, KNN, L.R., and Ridge were suitable to be used as a model of the FSGC.

### 3.3. Particle Swarm Optimization (PSO) Parameters Optimization

Besides the parameters of the classifier, the alpha value (constant weight for trading off classifier performance), the number of particles, and iteration also needed more attention. Thus, to obtain their best numbers, PSO was computed by trying all possibilities. N and OM were simultaneously determined for each trial using the Ridge model, a simple model that can bear prediction with many outputs. Finally, the comparison of R^2^ and RMSE of the calibration set for all the different results allowed us to find the optimal values. The more the model is efficient, the more the value of the parameter is optimal. An efficient model should have R^2^ closer to 1 and RMSE value near 0.

**Figure 4:**
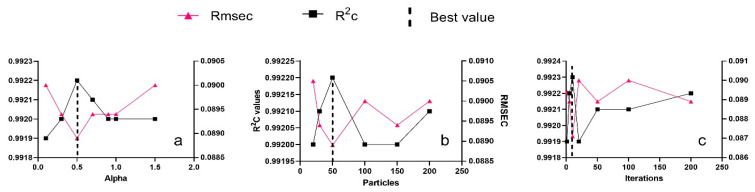
Best value for particle swarm optimization (PSO) by simultaneously predicting and comparing N and OM results using Ridge for calibration set; (**a**) alpha; (**b**) particles; (**c**) iterations.

In this experiment, the optimal alpha value was determined after trying different values (Figure 4a). The values of iteration and particle value have been initialized to 5 and 50 as default values. For each given value of alpha, N and O.M. were predicted simultaneously using the Ridge model. The lowest value of RMSEC and the highest value of R^2^c were found when alpha was initialized to 0.5 (Figure 4a). This point was used as the optimal value of alpha.

The best particle value was obtained among five different values (Figure 4b). Before predicting N and O.M., the alpha value was set to the obtained optimal value (0.5), and the iteration value has remained constant. The result has shown that the best particle value was 50 as it has the lowest RMSEC and the highest R^2^c.

Finally, different values of iteration were tried during the computation of PSO-FSGC. The results of the prediction of N and O.M. illustrated that the ideal iteration value is 10 as it has the minor values of RMSEC and the maximum value of R^2^c (Figure 4c).

### 3.4. Comparison between the Proposed Method with Lasso, Genetic Algorithm (GA), PSO-Support Vector Machine (SVM)

PSO-FSGC-Ridge, Lasso-Ridge, GA-Ridge, PSO-SVM-Ridge were computed to predict N and OM simultaneously. The best subset must have higher R^2^ and lower RMSE for calibration and prediction set. PSO-FSGC has obtained excellent results for the calibration set R^2^c = 0.9923, RMSEC = 0.087 and for prediction set R^2^p = 0.9892, RMSEP = 0.1. Good prediction was also obtained using PSO-SVM and GA for both calibration (R^2^c = 0.9920, 0.9922 and RMSEC = 0.089, 0.088) and prediction set (R^2^p = 0.9986, 0.9890 and RMSEP = 0.103, 0.101). However, lasso has obtained slightly lower results compared to other previous techniques for both calibrations set (R^2^c = 0.9835, RMSEC = 0.128) and prediction set (R^2^p = 0.9767, and RMSEP = 0.148).

**Figure 5:**
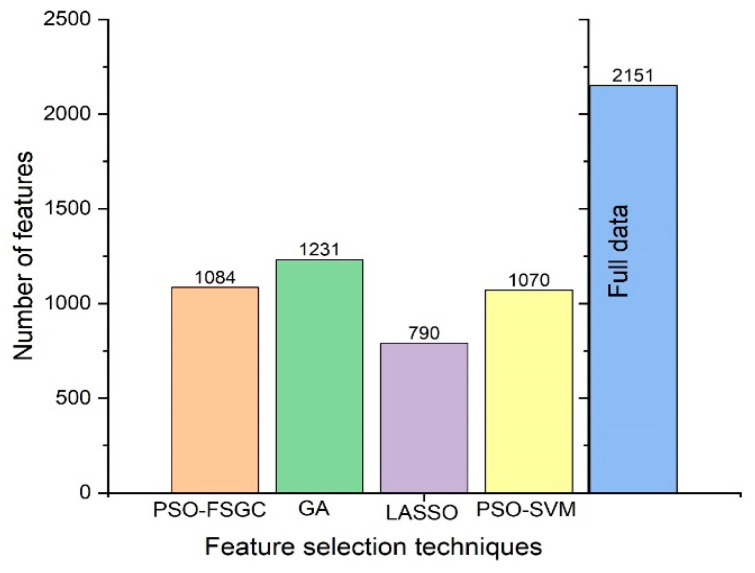
The number of feature variables of each selection technique.

Overall, the PSO-FSGC method outperformed all three techniques and has been shown to be efficient for finding the best subset. Figure 5 displays the number of feature variables of each selection technique.

### 3.5. Prediction of Nitrogen (N) and Organic Matter (OM) Using Second Stacked Generalizations for Regression (SSGR)

The Ridge method was used during the whole process because it considers several outputs (multi-output regressor) and simultaneously predicts N and OM. However, in this step, a new multiple-stacked generalization model was built to improve the prediction results. Compared to the first stacked generalization (FSGC) used as the PSO classifier, the second multiple-stacked generalization was used for regression purposes (SSGR).

All models and parameters used to build FSGC were used for SSGR except LR, only applicable when the dependent variable is dichotomous (binary). Therefore, it has been replaced by MLP. The samples were scaled using the standardization method (strategy for scaling data in which the values are centered around the mean with a unit standard deviation) and split into 607 samples for the calibration set and 203 samples for the prediction set using cross-validation. To check the reliability of the model, it has been compared with traditional methods such as Ridge, support vector machine for regression (SVR), and partial least square (PLS). The SVR computation was undertaken using the best parameter obtained after the grid search. The PLS model was computed with different latent variables (Lv = 3, Lv = 6, and Lv = 9) using the entire dataset.

#### 3.5.1. Prediction of Nitrogen (N)

**Figure 6:**
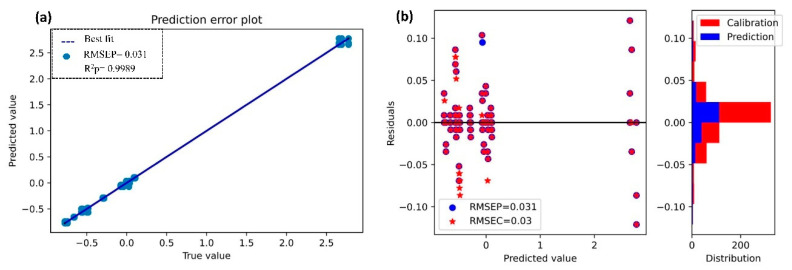
Determination of nitrogen (N) through second stacked generalizations for regression (SSGR). (**a**) Prediction error plot; (**b**) residual plot and distribution.

The results of the models for N with the selected bands obtained by PSO-FSGC are listed in Table 2. The best prediction for N was obtained when using the SSGR model for both calibrations set (R^2^c = 0. 9990, and RMSEC = 0.03) and prediction set (R^2^p = 0. 9989, and RMSEP = 0.031). Figure 6 shows the prediction error and the residuals plot of SSGR. There is a strong correlation between the predictions and their true values (Figure 6a). Moreover, a fairly random, uniform distribution of the residuals against the target in two dimensions (Figure 6b) can be noticed. This seems to indicate that our model is performing well.

#### 3.5.2. Prediction of Organic Matter (OM)

**Figure 7:**
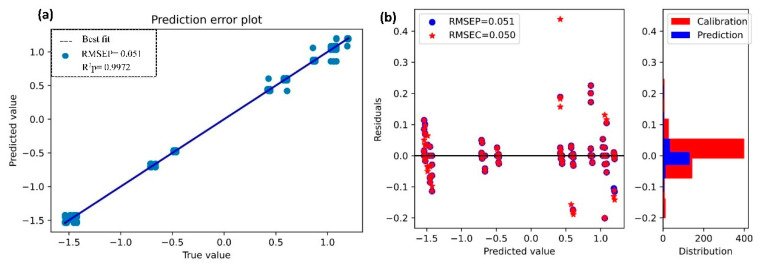
Determination of OM through SSGR. (**a**) Prediction error plot; (**b**) residual plot and distribution.

The OM values predicted using the four models with the selected bands obtained through the computation of PSO-FSGC are shown in Table 2. Among these models, SSGR produced an excellent result for calibration (R^2^c = 0. 9973, and RMSEC = 0.050) and prediction (R^2^p = 0. 9972, and RMSEP = 0.051). As for the prediction of N, it can be seen in Figure 7a that the prediction error also showed a strong correlation between predicted and true value. Moreover, the distribution of their difference demonstrated the good performance of SSGR.

## 4. Discussion

This study proposed a novel approach for predicting and evaluating the amount of organic matter (OM) and nitrogen (N) in organic fertilizer using Vis-NIR. However, the extensive features of the Vis-NIR spectra can make the prediction hard. Thus, to obtain the best subset, we created a new ensemble model (FSGC), which is used as the PSO classifier. The proposed approach outperformed other feature selection strategies such as PSO-SVM, GA, and Lasso in terms of prediction outcomes for both prediction and calibration sets. This success could be attributed to the constructed classifier, which is made by combining several models (Figure 3). Many previous studies have shown that good predictions could be obtained using GA or PSO-SVM [28,29,30]. In this case, despite the difference being barely noticed, PSO-FSGC outperformed PSO-SVM.

**Figure 8:**
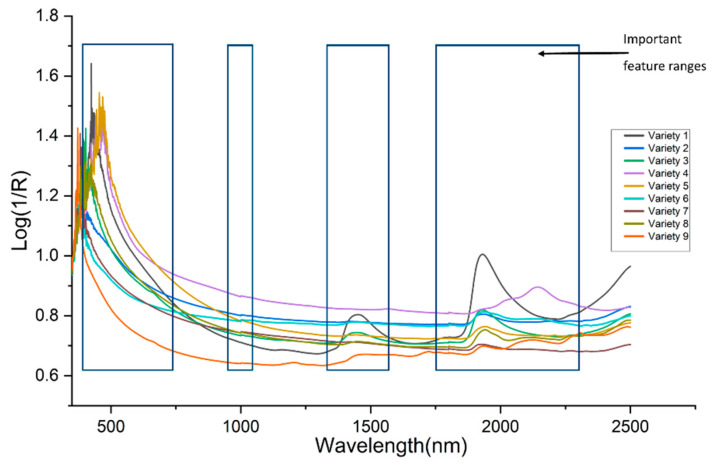
Average spectra of nine varieties and important features detected with PSO-FSGC.

High-ranking features are concentrated in the 400–600 nm, 900–1000 nm, 1400–1600 nm, and 1800–2300 nm ranges (Figure 8). According to Reda et al. [31], the wavelength at 1400 nm could be associated with the vibration of OH and residual water in organic matter, while the spectrum field at 2130 nm could be related to a range of vibration forms, including a stretch of C–H, deformation of N–H, and the stretch combination C=O; N–H. In this experiment, the large part of PSO-FSGC selected variables were between 1400–1600 nm and 1800–2300 nm and, according to Leme et al. [32], the band 1386–1401 nm may contribute to the prediction of organic matter. Furthermore, the features from range 400–600 nm and 1800–2300 nm could contribute widely to the prediction of N, which has been demonstrated in [33].

However, although Lasso gave the smallest number of selected variables (Figure 5), its prediction results were lower than the other three methods, which means that the number of variables chosen can affect the outcome. In other words, this finding could be explained by the fact that removing too many variables from the spectra can affect the prediction results.

Overall, the use of PSO-FSGA selected features is feasible to predict N and OM as it gives more insight into the spectral predictive mechanisms and can be suited for more efficient computation and storage [34].

Four machine learning methods were combined with PSO-FSGC to predict OM and N. Based on R^2^ and RMSE values, the SSGR achieved the highest performance in both calibration and prediction. At the same time, Ridge outperformed SVR and PLS slightly. The successful prediction performance of the SSGR might be the consequence of the ensemble models that were optimized with parameter tuning. It could also be the outcome of the proposed method (FSGC-PSO), which deleted some irrelevant features.

However, it could be noticed that the prediction results for N were barely more significant than the prediction results of OM. This result could be interpreted by the high number of features obtained from the 1800–2300 nm range, which contribute widely to N detection (Figure 8).

The scatterplots of the predicted vs. true value and the residual plots illustrated in Figure 6 and Figure 7 showed that the model performed well. A strong correlation between the prediction values (N and OM) and their actual values was observed in Figure 6a and Figure 7a. This strong correlation could explain the robustness of our model to predict N and OM.

Finally, it could be seen that the points were overlapped with each other, which is due to the small number of varieties used in this experiment

Although FSGC-PSO coupled with SSGR achieved excellent N and OM results, more samples with different varieties should be used in the future. Overall, Vis-NIR and the framework can play a crucial role in chemical components detection.

## 5. Conclusions

This study proposed a novel framework that combined a particle swarm optimization (PSO) and two multiple-stacked generalizations to determine the amount of organic matter (OM) and nitrogen (N) in organic fertilizer. The first multiple-stacked generalizations coupled with PSO (FSGC-PSO) were used to select the best features, while the second multiple-stacked generalizations (SSGR) improved the detection of N and OM. Compared to other feature selection approaches such as GA, Lasso, and PSO-SVM, PSO-FSGC was shown to be an effective method for finding the best subset. Meanwhile, the obtained FSGC-PSO subset combined with SSGR achieved more significant prediction results than conventional methods (Ridge, SVM, and PLS model) for the prediction of N (R^2^p = 0.9989, RMSEP = 0.031 and LOD = 2.97) and O.M. (R^2^p = 0.9972, RMSEP = 0.051 and LOD = 2.97). Therefore, this developed approach can be implemented as a promising way to determine N and O.M. in organic fertilizer, which has enormous benefits for rapid detection of chemical composition.

## Figures and Tables

**Table 1:** The statistics of organic matter (OM) and nitrogen (N) concentrations in samples.

Propriety	Variety	1	2	3	4	5	6	7	8	9
N	Min.	1.460	2.030	1.540	1.220	2.110	1.350	2.220	1.220	5.190
	Max.	1.480	2.070	1.560	1.260	2.160	1.360	2.240	5.330	5.330
	Mean	1.470	2.047	1.550	1.237	2.140	1.353	2.230	2.107	5.250
	SD	0.010	0.020	0.010	0.020	0.026	0.005	0.010	1.117	0.072
OM	Min.	55.97	40.23	40.56	58.15	57.11	47.12	54.12	40.23	45.65
	Max.	56.12	40.34	40.98	58.22	57.46	47.29	54.33	58.22	45.98
	Mean	56.02	40.30	40.78	58.19	57.29	47.22	54.25	50.21	45.78
	SD	0.083	0.058	0.210	0.035	0.175	0.090	0.111	6.348	0.175

**Table 2:** Prediction results of SSGR, Ridge, support vector machine for regression (SVR), and partial least square (PLS) for the determination of nitrogen (N) and organic matter (OM).

Proprieties Methods		Calibration		Prediction		
		R^2^c	RMSEC	R^2^p	RMSEP	LOD
N	SSGR	0.9990	0.030	0.9989	0.031	2.97
	Ridge	0.9987	0.036	0.9987	0.036	2.77
	SVR	0.9954	0.066	0.9951	0.068	2.81
	PLS (Lv = 3)	0.9790	0.143	0.9782	0.150	3.04
	PLS (Lv = 6)	0.9939	0.076	0.9934	0.082	2.99
	PLS (Lv = 9)	0.9985	0.038	0.9984	0.038	2.98
OM	SSGR	0.9973	0.050	0.9972	0.051	2.97
	Ridge	0.9858	0.011	0.9796	0.137	3.03
	SVR	0.9955	0.067	0.9945	0.071	3.00
	PLS (Lv = 3)	0.4953	0.708	0.4683	0.716	6.01
	PLS (Lv = 6)	0.9641	0.190	0.9516	0.213	3.09
	PLS (Lv = 9)	0.9829	0.131	0.9755	0.151	3.03

*R* ^2^ *c: Coefficient of determination for calibration; R* ^2^ *p: Coefficient of determination for prediction; RMSEC: Root mean square error for calibration; RMSEP: Root mean square error for prediction; LOD: Limit of detection.*

## Data Availability

The data presented in this study are available on request from the corresponding author. The data are not publicly available due to privacy.
